# The Aggregation of *α*-Synuclein in the Dorsomedial Striatum Significantly Impairs Cognitive Flexibility in Parkinson’s Disease Mice

**DOI:** 10.3390/biomedicines12081634

**Published:** 2024-07-23

**Authors:** Jing Chen, Yifang Liu, Mingyu Su, Yaoyu Sun, Chenkai Liu, Sihan Sun, Ting Wang, Chuanxi Tang

**Affiliations:** 1 School of Basic Medical Science, Xuzhou Medical University, Xuzhou 221004, China; 2 Department of Neurobiology, Xuzhou Medical University, Xuzhou 221004, China; 3 The Second Clinical Medical College, Xuzhou Medical University, Xuzhou 221004, China; 4 The Research and Engineering Center of Xuzhou Neurodegenerative Disease Diagnosis and Treatment Biologics, Xuzhou Medical University, Xuzhou 221004, China

**Keywords:** Parkinson’s disease, *α*-synuclein, acetylcholine, muscarinic receptor 1, reversal learning, cognitive flexibility

## Abstract

This study focused on α-synuclein (α-syn) aggregation in the dorsomedial striatum (DMS) so as to investigate its role in the cognitive flexibility of Parkinson’s disease (PD). Here, we investigated the cognitive flexibility by assessing reversal learning abilities in MPTP-induced subacute PD model mice and in C57BL/6J mice with α-syn aggregation in the DMS induced by adenovirus (AAV-SNCA) injection, followed by an analysis of the target protein’s expression and distribution. PD mice exhibited impairments in reversal learning, positively correlated with the expression of phosphorylated α-syn in the DMS. Furthermore, the mice in the AAV-SNCA group exhibited reversal learning deficits and a reduction in acetylcholine levels, accompanied by protein alterations within the DMS. Notably, the administration of a muscarinic receptor 1 (M1R) agonist was able to alleviate the aforementioned phenomenon. These findings suggest that the impaired cognitive flexibility in PD may be attributed to the diminished activation of acetylcholine to M1R caused by α-syn aggregation.

## 1. Introduction

Parkinson’s disease (PD) is a neurodegenerative disorder primarily characterized by motor impairment alongside various non-motor symptoms, with a notable increase in patient prevalence observed in recent years [1]. Studies indicate that cognitive dysfunction affects approximately 40% of PD patients, often preceding motor symptoms [2]. Cognitive impairment in PD, particularly executive dysfunction, is frequently associated with diminished cognitive flexibility [3]. This decline manifests prominently as deficits in reversal learning, impairing the individual’s capacity to plan, organize, and adapt goal-directed behavior in response to environmental changes [4,5]. Understanding the underlying mechanisms of reversal learning deficits in PD not only offers insights into non-motor aspects of the disease but also provides a foundation for developing therapeutic strategies targeting cognitive impairment. Despite the recognized importance of the dorsomedial striatum in reversal learning regulation, the specific involvement of its neuronal subtypes remains unclear. While optogenetic studies suggest a role for dopamine D2 receptors on indirect pathway spiny projection neurons (iSPNs), clinical treatment with levodopa fails to ameliorate reversal learning deficits, indicating potential complexities in the mechanism [6]. Consequently, there is a need to explore alternative perspectives to elucidate the contribution of iSPNs to the reversal of learning deficits in PD.

According to the literature, the reversal learning process is associated with a notable rise in acetylcholine (ACh) levels within the striatum [7,8]. Despite constituting only 3–4% of striatal neurons, cholinergic interneurons (ChI) serve as the primary source of ACh in this brain region [9,10]. Studies demonstrate that ChI receives and integrates glutamatergic inputs from the parafascicular nucleus of the thalamus, contributing to reversal learning regulation [11,12]. Furthermore, thalamic parafascicular nucleus inactivation during reversal learning impedes ACh release in the dorsomedial striatum, resulting in decreased reversal learning ability [13]. Muscarinic ACh receptor 1 (M1R) expression on indirect pathway dopamine receptor D2 (DRD2) spiny projection neurons (iSPNs) allows ACh to modulate iSPN synaptic plasticity through M1R activation, enhancing iSPN excitability [14]. Pharmacological inhibition of M1R in the dorsomedial striatum disrupts reversal learning in rats [15].

One of the principal pathological features of PD is the extensive accumulation of α-synuclein (α-syn) in the nigrostriatal pathway, forming Lewy bodies [16]. Clinical evidence indicates significant cholinergic depletion and co-localization of cholinergic fibers and α-syn aggregates in PD patients with cognitive impairment [17]. Previous research demonstrates that α-syn aggregation inhibits neuronal activation by reducing surface N-methyl-D-aspartate receptor (NMDAR) distribution [18]. Electrophysiological studies suggest that α-syn aggregation weakens ChI activation without affecting spiny projection neurons (SPNs), attributed to distinct NMDAR subunits NR2D and NR2B in ChI and SPNs, respectively, [19]. Consequently, we hypothesize that α-syn aggregation in the dorsomedial striatum may diminish M1R activation on iSPNs by impeding ChI activation and ACh release, ultimately contributing to reversal learning deficits.

Our study investigated the impact of α-synuclein accumulation in the substantia nigra pars compacta-striatum pathway on the suppression of cholinergic neuron activity, subsequently leading to a reduction in Ach levels within the striatal region. This cascade of events triggered molecular alterations associated with D2-iSPN activity and synaptic plasticity. Consequently, in PD mice, this disruption manifested as impaired performance in cognitive flexibility tasks, particularly in reverse learning tests.

## 2. Materials and Methods

### 2.1. Mice

This study utilized 8–10-week-old male C57BL/6J mice (SPF grade, weighing 25 ± 3 g), sourced from GemPharmatech Co.,Ltd. (Nanjing, China) (License No.: SYXK (Su) 2018-0008; Certificate No.: NO. 202137412). The mice underwent testing one week after acclimatizing and breeding at our school’s animal center. Housing conditions included individual ventilation systems, maintaining a temperature of 22–24 °C, humidity between 40 and 60%, and a 12/12 h light/dark cycle. Standard feed and corn cob bedding were procured from Jiangsu Xietong Pharmaceutical Bio-engineering Co., Ltd. (Nanjing, China) and Pizhou Xiaohe Technology Development Co., Ltd. (Xuzhou, China), respectively. All animal study was approved by and performed in accordance with the guidelines of the IACUC of Xuzhou Medical University (Experimental Animal Ethics No.: 202209S042).

Thirty-five normal male C57BL/6J mice were randomly assigned to the control and MPTP groups. Additionally, 25 C57BL/6J mice were randomly divided into three groups: control, AAV-NC, and AAV-SNCA. Furthermore, 25 C57BL/6J mice were selected for DMSO or VU0357017 (20 mg/kg; Cat# T3619, TargetMol Chemicals Inc., Boston, MA, USA) injections and randomly assigned to three groups: AAV-NC + DMSO, AAV-SNCA + DMSO, and AAV-SNCA + VU0357017.

### 2.2. MPTP Subacute Modeling

Following intraperitoneal administration of 1-methyl-4-phenyl-1,2,3,6-tetrahydropyridine (MPTP), astrocytes oxidize it into the potent dopaminergic neurotoxin, 1-methyl-4- phenylpyridinium ion (MPP+). This compound is subsequently absorbed by dopaminergic neurons, triggering apoptosis and leading to the pathological changes observed in Parkinson’s disease (PD). To establish a PD mouse model, this study employed the subacute MPTP modeling adaptability of acetylcholine towards reversal learning, highlighting the role played by M1R agonists in facilitating this process only under conditions of reduced acetylcholine levels (7 mg/mL; Cat# M0896, Sigma, St. Louis, MO, USA) at a dose of 30 mg/kg administered over five consecutive days.

### 2.3. Stereotaxic Intracranial Injection

First, mice were weighed, and then sodium pentobarbital was administered intraperitoneally at a dose of 45 mg/kg to induce anesthesia. After ensuring the mice were adequately anesthetized, necessary instruments were prepared, and the microinjector was securely fixed onto the injection pump of the brain stereotaxic apparatus. The required volume of the drug was injected into the striatum (AP: +0.5 mm, ML: +1.25 mm, DV: −2.5 mm; refer to The Mouse Brain in Stereotaxic Coordinates, Second Edition). Injection commenced at a controlled rate of 0.25 μL/min, and following completion, the needle was left in place for 5 min to facilitate proper diffusion of the drug. Mice designated for brain stereotactic injection were subjected to the aforementioned procedures. In accordance with the literature recommendations, an injection volume of 0.5 μL of adeno-associated virus was administered.

### 2.4. Rotarod Test

During the adaptation period lasting three days subsequent to modeling, the mice underwent daily training sessions to facilitate acclimatization to the experimental conditions. Each day’s training session consisted of a single session. Moving to the testing period, on the designated assessment day, mice were initially subjected to a ten-minute adaptation and training phase at a rotational speed of 30 revolutions per minute (r/min). Subsequently, they were allowed a ten-minute rest period before the commencement of testing. The actual testing duration spanned five minutes, during which the rotational speed gradually escalated from 30 to 40 r/min over a three-minute interval. Termination criteria for the test involved instances where mice either fall from the rod or engage in continuous rotation for three consecutive rounds, prompting immediate removal from the apparatus and conclusion of the test session (ZH-600, Zheng Hua Tech. Co., Ltd., Hefei, China). Data recording encompassed the duration of time each mouse remained on the rod and the rotational speed at the point of falling.

### 2.5. Open-Field Test

The movement of mice was recorded in an open-field test. The field was divided into 16 squares (4 center, 12 peripheral) using ANY-maze software 7.40 (Stoelting Co., Wood Dale, IL, USA). After a 30 min acclimatization period, the mice were placed in the center and their behavior was recorded for 5 min (the box was disinfected after each test). Motor performance was evaluated based on the total distance covered. The ANY-maze software accurately tracked and analyzed mouse behavior to provide precise data.

### 2.6. Modified Morris Water Maze

The Morris water maze (ZH-SBS, Zheng Hua Tech. Co., Ltd., Hefei, China) can test the cognitive function of animals. This study refers to the water maze detection method used by David Thonnard et al. [20] to observe the latency of mice to find the platform after the platform is switched, and the proportion of time spent in each quadrant after the platform is removed, to evaluate the reversal learning ability of mice.

### 2.7. Small Animal Touchscreen Behavioral System—Visual Discrimination Reversal

**Figure 1:**
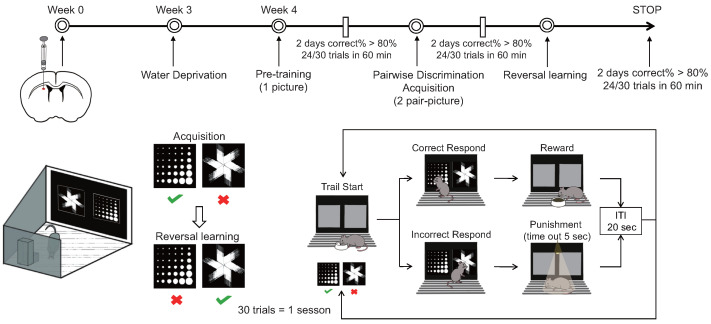
Mode diagram of small animal touch screen system.

A Bussey–Saksida touchscreen chamber was used to test cognitive flexibility (Figure 1). (a) During the water thirst period spanning from the first to the fifth day, mice had their access to drinking water progressively restricted. The allotted drinking time diminished successively over six intervals: 20 h, 12 h, 8 h, 4 h, and 2 h, culminating in a final period of two hours of free access to water daily during the touch screen behavior experiment. If decreases in weight or changes in mental state occurred among the mice, the experiment was promptly halted and reinstated after the reintroduction of water. (b) The adaptive training phase, spanning four days, commenced with mice being introduced to the test environment devoid of stimuli, followed by a 10 min acclimation period on the first day. Subsequently, the mice were exposed to the test environment with the illumination of a light on the food tray accompanied by an auditory cue and the provision of a 150 μL reward of sugar water (1% cane sugar water). Upon entry into the tray, mice received the reward, after which the tray light was extinguished and the auditory cue ceased. The training duration gradually extended to 20, 30, and 40 min on the second, third, and fourth days, respectively. (c) Initial touch training, conducted over one day, involved placing mice in the test environment with two illuminated screens, one displaying a random image while the other remained blank for a duration of 30 s. Upon illumination of the tray light accompanied by an auditory cue, mice received a 7 μL reward of sugar water upon touching the image. Subsequent images were presented every 20 s. If mice touched the image, they were rewarded with 21 μL of sugar water. This training session lasted for 60 min, encompassing a total of 30 trials. (d) Pair discrimination training commenced by presenting mice with two illuminated screens, one displaying a random image and the other remaining blank for 30 s, during which mice were rewarded for touching the image and received no reward for touching or failing to touch the blank screen. Over five days of continuous training, failure to touch or touching a blank screen resulted in no reward and was illuminated as punishment. Upon achieving a correct response rate of 77% within 30 min for two consecutive days (23/30 trials), mice progressed to the subsequent stage. (e) The paired discrimination test was initiated by illuminating the tray light and dispensing sugar water rewards, prompting mice to touch the tray and commence training. Two different images were concurrently presented, with one designated as correct. Upon touching the correct image, mice were rewarded with sugar water; however, touching the incorrect image elicited a light punishment. Mice were required to attain an accuracy rate of 80% within 60 min for two consecutive days (24/30 trials) before advancing to the subsequent stage. (f) The reverse learning test involved setting the previously incorrect image as the correct one, offering sugar water rewards upon its selection. Mice failing to choose the correct image received a light punishment. To conclude the test, mice needed to achieve an accuracy rate of 80% within 60 min for two consecutive days (24/30 trials). Data encompassing the time taken, number of trials, and accuracy rate attained by mice in both the paired discrimination and reverse learning tests were recorded and analyzed to evaluate the mice’s reversal learning ability through statistical means.

### 2.8. Extraction of Total Protein from Dorsomedial Striatum

Upon cervical dislocation and subsequent demise of the mice, the scalp and skull were promptly removed, and the brain tissue was dissected followed by immersion in pre-cooled PBS solution to facilitate gentle rinsing, eliminating residual blood and impurities. The dissected dorsomedial striatum was meticulously isolated with reference to the brain atlas and transferred into labeled 2 mL centrifuge tubes for storage at −80 °C. Subsequently, the samples were placed on pre-prepared ice for stabilization and weighed, following which 400 μL of lysis buffer (RIPA) (P0013, Beyotime, Shanghai, China) and 4 μL of PMSF (ST2573, Beyotime, Shanghai, China) were added per 100 mg of sample weight. Homogenization of the samples ensued using a tissue homogenizer, with intermittent halts every three seconds to mitigate the risk of overheating and protein degradation. Prior to homogenizing different samples, the homogenizer was rinsed with pre-cooled phosphate-buffered saline (PBS) (B040100, Sangon Biotech, Shanghai, China), and excess liquid was absorbed with filter paper before subsequent homogenization. Post-homogenization, samples were allowed to lyse on ice for 5 min, followed by vortexing, with this process iterated three times at 5 min intervals. The centrifuge was pre-cooled to 4 °C, and the lysed tissue was centrifuged at 14,000 rpm for 20 min. The supernatant obtained post-centrifugation was carefully collected and stored at −80 °C for further analysis.

### 2.9. BCA Method for Protein Concentration Detection and Protein Concentration Leveling

The BCA working solution was prepared in accordance with the instructions provided in the BCA kit, with BCA reagents A and B mixed at a ratio of 50:1, tailored to the number of samples intended for analysis. Subsequently, a 96-well plate was sequentially loaded with varying volumes of the prepared protein standard solution (ranging from 0 to 20 μL) to achieve protein concentrations of 0, 0.025, 0.05, 0.1, 0.2, 0.3, 0.4, and 0.5 mg/mL in each well, followed by addition of 2 μL of the sample to be tested and double-distilled water to reach a total volume of 20 μL per well. The BCA working solution (200 μL) was then added to all wells, and the plate was gently mixed before incubating in a 37 °C incubator for 30 min. Subsequently, the multifunctional microplate reader (Synergy H1, BioTek, Hercules, CA, USA) was activated, preheated to 37 °C, and configured with appropriate parameters to measure the optical density (OD) value of each well at a wavelength of 562 nm. Protein concentrations of the sample wells were determined by reference to the standard curve obtained. Furthermore, the absorbance OD value at a wavelength of 562 nm was measured using the multifunctional microplate reader preheated to 37 °C, and the protein concentration of each sample was calculated based on the standard curve. Finally, RIPA and protein loading buffer volumes required for standardization were determined based on the protein concentration, sequentially added and mixed, followed by denaturation of the protein at 100 °C for 10 min in a constant temperature mixer and subsequent cooling to room temperature for storage at −20 °C.

### 2.10. Western Blotting (WB)

Prepare 1.5 mm glass plates and corresponding 10-well or 15-well combs by washing with double-distilled water and drying in an incubator. Align and insert the glass plates into the plastic gel frame, ensuring tight clamping and absence of gaps to prevent gel leakage. Prepare the separating gel pre-solution as instructed, pour it between the glass plates, avoiding bubble formation, and gently add 75% ethanol to level the gel surface and remove excess bubbles. After 20 min, remove excess ethanol, prepare the stacking gel pre-solution, pour it between the glass plates, and insert the comb. Allow 20 min for the stacking gel to solidify. Transfer the solidified gel plate to the electrophoresis device slot, fill with electrophoresis solution, remove the comb, and pipette excess gel from the sample wells. After boiling and cooling the samples, load them into the wells along with protein marker. Set the electrophoresis apparatus to 80 V initially, then adjust to 120 V when the Marker reaches the separating gel bottom. For electrotransfer, cut out the target protein area from the gel, and soak the gel strip and a corresponding size NC membrane in electrotransfer solution. Assemble sponge pads, filter papers, gel strip, NC membrane, and pads into an electrotransfer clip, ensuring no bubbles between layers. Transfer to the electrotransfer device with an ice box, add electrotransfer solution, set to 300 mA constant current, and run for the time equivalent to the target protein molecular weight plus 20 min.

Remove the NC membrane after electrotransfer, mark it, and immerse in 5% skim milk. Incubate on a shaker at room temperature for 2 h. Subsequent to blocking, transfer the NC membrane to washing buffer for three 5 min washes. Then, incubate the NC membrane overnight at 4°C in the prepared primary antibody solution. Following primary antibody incubation, transfer the NC membrane to washing buffer for three 5 min washes. Subsequently, based on the host source property of the primary antibody, incubate the NC membrane in the corresponding prepared secondary antibody solution on a shaker at room temperature for 2 h in the dark. After secondary antibody incubation, wash the NC membrane three times in washing buffer under dark conditions for 5 min each. Proceed to scan the membrane using the Odyssey dual-color infrared laser imaging scanner (Odyssey CLX, LI-COR, Inc., Lincoln, NE, USA), ensuring bubble-free placement. Finally, analyze the results using Image J software for grayscale analysis, and organize and record the findings for statistical analysis.

### 2.11. Enzyme-Linked Immunosorbent Assay (ELISA)

We used an ELISA kit (Mouse Acetylcholinesterase ELISA Kit, JL20661-48T, Jianglai industrial Limited By Share Ltd., Shanghai, China). First, standard and sample wells were designated, with 50 μL of various standard concentrations meticulously added to each standard well. Following this, the sample loading process involved allocating blank control wells and wells designated for tested samples. To the latter, 40 μL of sample diluent was meticulously added, followed by 10 μL of the sample itself, achieving a final sample dilution factor of 5. Enzyme addition followed suit, with 100 μL of enzyme-labeled reagent dispensed into each well, excluding the blank wells. Following sealing of the plate with a film, incubation ensued at 37 °C for 60 min. Subsequent steps involved the preparation of washing solution, washing of the plate, color development, termination of the reaction, and final measurement of absorbance at 450 nm wavelength with a multifunctional microplate reader (Synergy H1, BioTek, Hercules, CA, USA), with particular emphasis on ensuring measurements were conducted within 15 min post-addition of the stop solution.

### 2.12. Tissue Fixation and Frozen Section

Upon anesthesia with Isoflurane (induced 3–4% concentration, Maintained at 1–1.5% concentration) (RWD Life Technology Co., Shenzhen, China), the mouse’s limbs were secured onto the perfusion board for stability. Subsequently, a small incision was made beneath the xiphoid process of the sternum using surgical scissors. The incision extended along the abdominal midline and sternal xiphoid midline to the mandible, with meticulous separation of subcutaneous tissue and flipping of the skin to expose the internal anatomy. The sternum was incised along the abdominal and sternal midlines, extending along the diaphragm to ensure full heart exposure. Hemostatic forceps were employed to tightly secure the sternum and chest skin, facilitating outward flipping of the forceps to fully expose the heart, followed by careful pericardium opening with tweezers. Subsequently, the perfusion needle was inserted into the left ventricle of the mouse heart approximately 3–5 mm from the apex, and the right atrium was incised for the rapid infusion of pre-cooled saline until the liver exhibited whitening or clear liquid was observed flowing from the right atrium. Following this, approximately 50 mL of pre-cooled 4% paraformaldehyde solution was administered, initially injected rapidly followed by a slower infusion. Upon completion of perfusion, the mouse brain tissue was dissected, immersed in 4 °C paraformaldehyde for 6–8 h, and subsequently transferred to 20% sucrose solution for dehydration. Once the brain tissue settled at the bottom of the centrifuge tube, it was replaced with 30% sucrose solution, paving the way for subsequent procedures.

The cryostat chamber and freezing head were adjusted to a temperature of −20 °C (CM1950, Leica, Wetzlar, Germany). Subsequently, the dehydrated brain tissue was carefully removed and excess surface liquid was absorbed using filter paper. Using a blade, the brain tissue was meticulously trimmed before embedding it onto the freezing table within the cryostat chamber using an embedding agent. Following complete solidification of the embedding agent, the tissue was secured onto the freezing head. Section thickness was set to 20 μm, and the target brain region was identified and retained based on reference to the brain atlas and optical microscope observation. The selected tissue sections were affixed onto slides, air-dried at room temperature, and stored at −20 °C for subsequent analysis.

### 2.13. Immunofluorescence (IF)

The frozen section samples were retrieved from −20 °C and allowed to equilibrate at room temperature for 30 min. Subsequently, the section samples were immersed in PBS solution and subjected to three washes, each lasting 5 min. Following this, the samples were air-dried and outlined with a histochemical pen. The prepared blocking solution was then applied, ensuring complete coverage of the sample, and left to incubate at room temperature for 2 h. Upon completion of blocking, excess blocking solution was removed, and the samples were exposed to the prepared primary antibody solution, followed by an overnight incubation at 4 °C. Afterward, the section samples were brought back to room temperature for 30 min, the primary antibody solution was removed, and the samples were washed three times in PBS solution, each wash lasting 5 min. Subsequent to removing excess liquid with filter paper, the prepared fluorescent secondary antibody solution was applied in darkness, with incubation at room temperature for 2 h. Following removal of the secondary antibody solution, the samples underwent another round of washing with PBS solution three times, each for 5 min. Subsequently, excess liquid was absorbed, and DAPI staining solution was added in darkness, with incubation at room temperature for 5–10 min. Following removal of the DAPI staining solution, the samples were washed three times with PBS solution, each wash lasting 5 min. Finally, anti-fluorescence quenching agent was used for sealing, and the fluorescence results were observed under an upright fluorescence microscope (BX-43, Olympus, Tokyo, Japan). Images were captured and saved, followed by fluorescence intensity analysis using Image J 1.8.0 software (National Institutes of Health, Bethesda, MD, USA).

### 2.14. Statistical Analysis

SPSS 22.0 statistical analysis software (IBM SPSS Inc., Chicago, IL, USA) was used to perform statistical analysis on the experimental data obtained. Quantitative data were expressed as mean ± standard deviation. Data comparison between two groups was performed using two independent sample *t*-tests. Single-factor comparison of data between multiple groups was performed using one-way analysis of variance (one-way ANOVA). Multiple factors were analyzed using repeated measures analysis of variance. Pearson correlation analysis was used to test the correlation between p-α-syn expression level and reversal learning score. *p* < 0.05 was considered statistically significant; statistical graphs were plotted using GraphPad 8.0.2 and Adobe Illustrator software 23.0.3 (Adobe, San Jose, CA, USA).

## 3. Results

### 3.1. The PD Mice Exhibit Deficits in Reversal Learning

**Figure 2:**
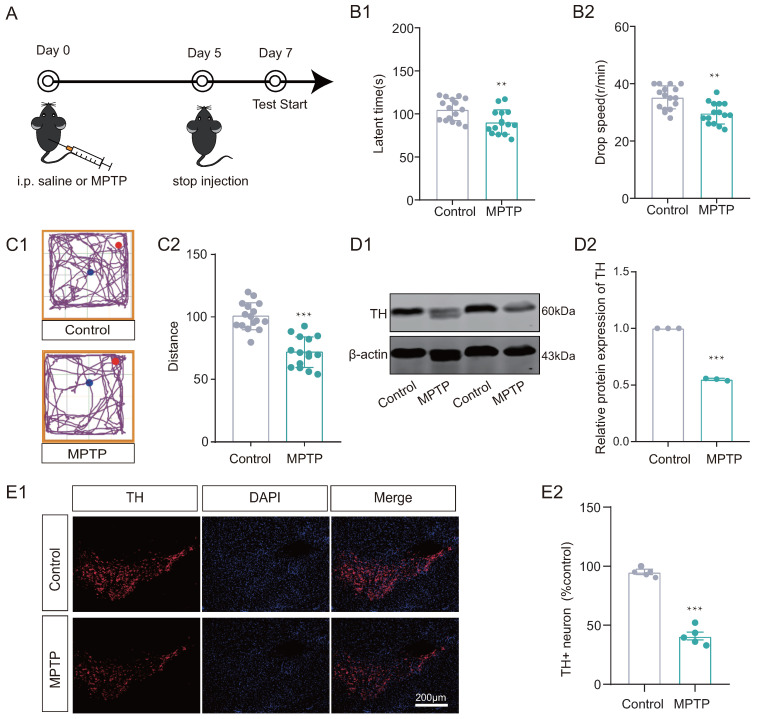
MPTP subacute Parkinson’s disease (PD) model was established. (**A**) The timeline for experimental arrangement. (**B1**,**B2**) Rotarod test and (**C1**,**C2**) Open-field test results suggested that the mice of MPTP group exhibited motor disability (*n* = 15–16 mice, ** *p* < 0.01, *** *p* < 0.001 vs. control group). (**D1**,**D2**) Western Blotting (WB) results for TH in substantia nigra, showed decreased expression in MPTP group (*n* = 3, *** *p* < 0.001 vs. control group). (**E1**,**E2**) Immunofluorescence (IF) results showed that the distribution of TH+ positive neurons decreased in MPTP group (*n* = 5, *** *p* < 0.001 vs. control group). Scale bar: 200 µm.

Initially, the MPTP subacute PD model mouse was developed to investigate whether PD mice display deficits in reversal learning abilities. We work on the following timeline (Figure 2A). Mice in the MPTP group exhibited impaired locomotor performance compared to the control group, as assessed by the Rotarod test and open-field test (Figure 2(B1,B2,C1,C2)). Additionally, decreased TH levels were observed in the substantia nigra of MPTP group mice using both Western blotting and immunofluorescence techniques (Figure 2(D1,D2,E1,E2)) compared to the control group. In addition, ELISA results showed that dopamine levels were significantly decreased in the substantia nigra of the MPTP group (see Supplementary Material S1).

**Figure 3:**
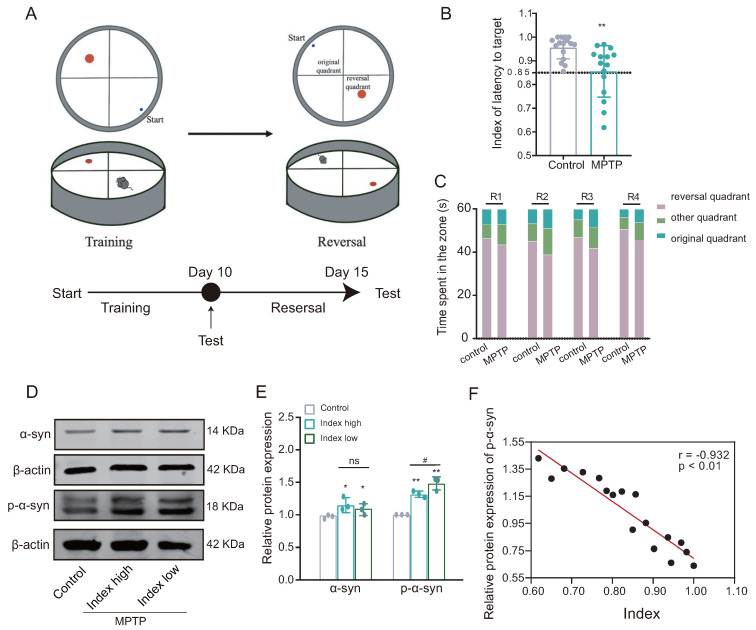
The PD mice demonstrate impaired reversal learning abilities associated with the accumulation of alpha-synuclein (α-syn). (**A**) Behavioral paradigm diagram of the modified Morris water maze (MWM). (**B**) The latency target index (referring to the time taken by the mice to reach the plateau quadrant for the first time during testing) serves as an indicator of reversal learning performance; low index indicates poor reversal learning in the MPTP group ((training test − reversal test)/(training test + reversal test); *n* = 15–16 mice, ** *p* < 0.01 vs. control group). The lower the index, the worse the ability in reversal learning. (**C**) Time spent in each quadrant by mice in the reversal learning phase showed that the MPTP group spent relatively more time in the original quadrant compared to the control group (*n* = 15–16 mice). (**D**,**E**) WB results for α-syn and p-α-syn in the dorsomedial striatum, showed increased expression in the low-index (index ≤ 0.85) group (*n* = 3; * *p* < 0.05, ** *p* < 0.01 vs. control group; # *p* < 0.05 vs. high group (index > 0.85)); while, the statistical difference in the levels of α-syn between the Index high and Index low groups was not significant. (**F**) Pearson correlation analysis of p-α-syn expression and reverse learning index (*n* = 17 mice, r = −0.932, *p* < 0.01).

Following the successful establishment of the PD mouse model, the reversal learning ability of PD mice was evaluated using a modified Morris water maze paradigm (Figure 3A). The reversal learning score, indicated by latency to reach the target, was significantly lower in the MPTP group compared to the control group (Figure 3B). Furthermore, during the reverse learning stage, mice in the MPTP group demonstrated a higher preference for the original quadrant compared to the control group (Figure 3C). These findings suggest impairment in reversal learning ability among mice in the MPTP group, as indicated by their lower reversal learning scores.

### 3.2. Reversal Learning Deficits in PD Mice Are Associated with Accumulation of α-Syn

Subsequently, based on the inverse learning score, the mice in the MPTP group were divided into two groups: high index (index > 0.85) and low index (index ≤ 0.85). Western blotting analysis showed that the expression of both alpha-synuclein (α-syn) and phosphorylated α-syn (p-α-syn) was significantly increased in the dorsal striatum of both subgroups of the MPTP group (low-index and high-index groups) compared with the control group; moreover, the expression of p-α-syn was also increased in the low-index group compared to the high-index group, while the difference in α-syn expression between the two groups was not statistically significant (Figure 3D,E). The Pearson correlation analysis revealed a statistically significant inverse relationship between the expression of p-α-syn and the score of reversal learning (Figure 3F), suggesting reversal learning deficits is positively correlated with α-syn aggregation (the lower the index, the worse the ability in reversal learning).

### 3.3. Induction of α-Syn Aggregation in the Striatum Leads to Reversal Learning Deficits

**Figure 4:**
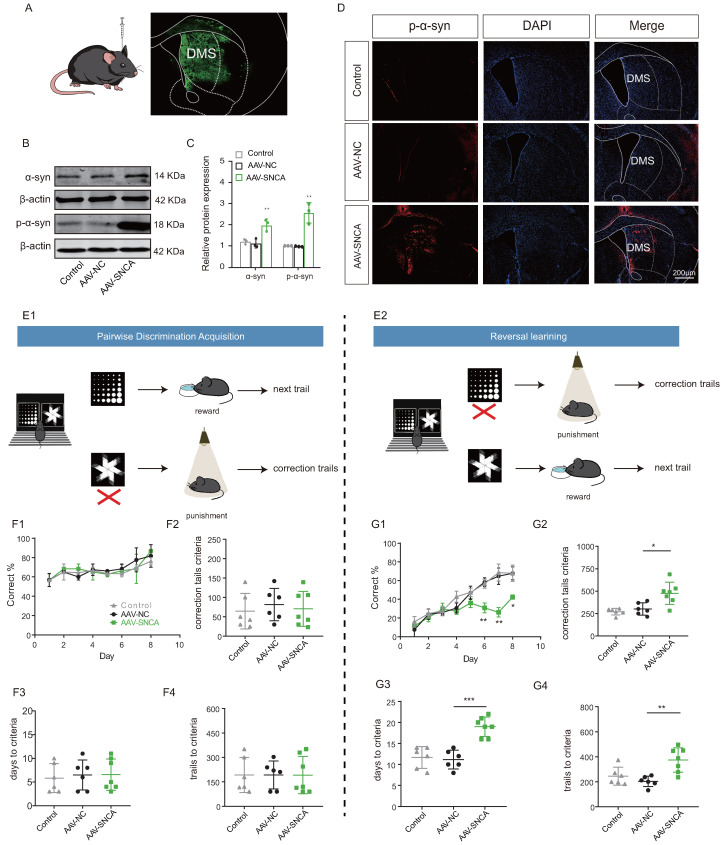
Reversal learning deficits were observed upon accumulation of α-syn in the dorsomedial striatum. (**A**) Schematic diagram of cerebral stereotactic drug delivery, and AAV-SNCA spread region in the striatum visualized in green. (**B**,**C**) WB results for α-syn and p-α-syn in the striatum showing increased expression in the AAV-SNCA group (*n* = 3, ** *p* < 0.01 vs. AAV-NC group). (**D**) IF results for striatal p-α-syn (scale bar: 200 μm). (**E1**,**E2**) Behavioral paradigm diagram of pairwise discrimination acquisition phases and reversal learning phases in the small animal touchscreen systems. (**F1**) Correctness per day for each group in the pairwise discrimination learning phase when 50% of the mice reach the criterion (>80% correct on two consecutive days). (**F2**–**F4**) The number of errors corrected, learning sessions and time required to achieve the criterion in the pairwise discrimination learning phase. (**G1**) Correctness per day for each group in the reversal phase when 50% of the mice reach the criterion. (**G2**–**G4**) The number of errors corrected, learning sessions and time required to achieve the criterion in the reversal phase. (*n* = 6–7 mice, * *p* < 0.05, ** *p* < 0.01, *** *p* < 0.001 vs. AAV-NC group.

The induction of adeno-associated virus (AAV-SNCA) was employed to further elucidate the correlation between aggregation of α-syn in the dorsomedial striatum and the ability of reversal learning (Figure 4A). Western blotting analysis (Figure 4B,C) showed an increase in the expression both of α-syn and p-α-syn in the striatum of mice injected with AAV-SNCA compared to those injected with AAV-NC; immunofluorescence staining (Figure 4D) gave also similar results for p-α-syn.

A small animal touch screen system for visual discrimination reversal test was employed to assess the mice’s ability in reversal learning (Figure 4(E1,E2)). During the paired discrimination learning phase, there were no statistically significant differences observed in correct rate, time to criterion (achieving 80% or more correct on two consecutive days), number of error corrections, and number of learning sessions among the three groups (Figure 4(F1–F4)). The initial learning ability of the mice remained unaffected by α-syn aggregation. However, during the reversal learning phase, mice in the AAV-SNCA group exhibited significantly lower correct rates on days 6-8 compared to the AAV-NC group (Figure 4(G1)). Additionally, the time to reach the criterion, the number of error corrections, and the number of learning sessions were significantly increased (Figure 4(G2–G4)). The findings suggest that α-syn aggregation in the dorsomedial striatum may contribute to deficits in reversal learning in mice.

### 3.4. The Aggregation of α-Syn in the Dorsomedial Striatal Hinders ACh Release from Cholinergic Interneurons (ChI)

**Figure 5:**
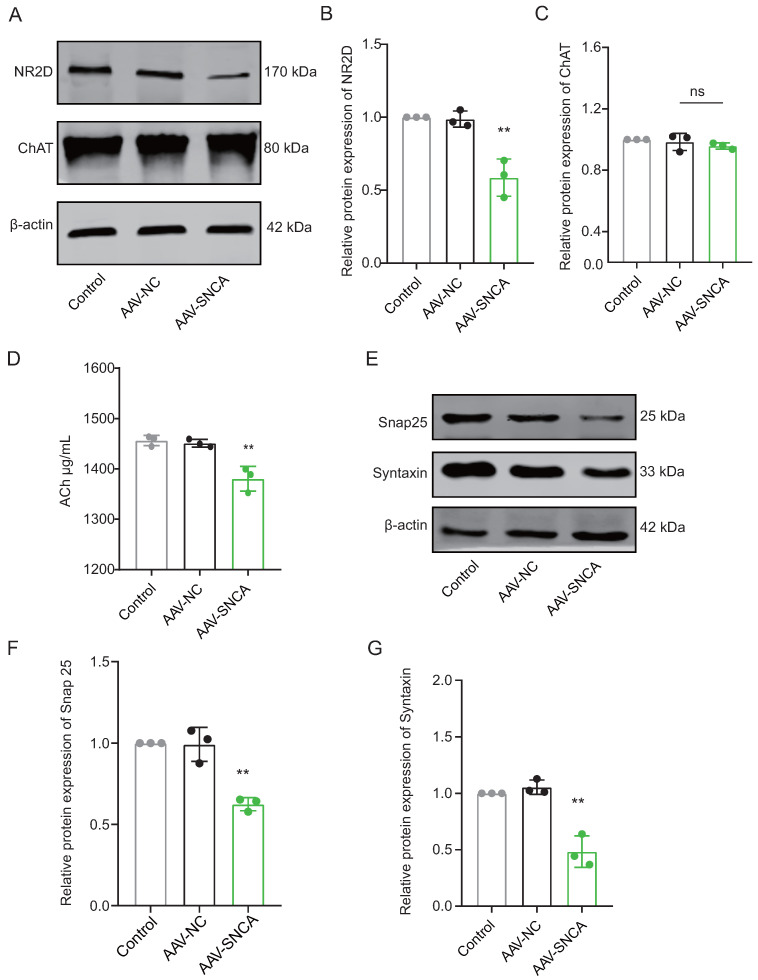
The aggregation of α-syn in the dorsomedial striatum inhibited acetylcholine (Ach) release from cholinergic interneurons (ChI). (**A**–**C**) WB results and statistical analysis of NR2D and Choline acetyl transferase (ChAT) in the dorsomedial striatum (*n* = 3, ** *p* < 0.01 vs. AAV-NC group); however, there was no statistically significant difference observed in the levels of ChAT between the AAV-NC group and the AAV-SNCA group. (**D**) ELISA results showed a decrease in ACh content in the dorsomedial striatum of mice in the AAV-SNCA group (*n* = 8, ** *p* < 0.01 vs. AAV-NC group). (**E**–**G**) WB results and statistical analysis of Snap25 and Syntaxin in the dorsomedial striatum (*n* = 3, ** *p* < 0.01 vs. AAV-NC group).

The process of reversal learning consistently correlates with a significant elevation in ACh levels within the dorsomedial striatum, primarily originating from cholinergic interneurons (ChI). The aggregation of α-syn can hinder neuronal activation by diminishing NMDA receptor (NMDAR) expression. Western blotting analysis revealed a significant decrease in the expression level of NR2D in the dorsomedial striatum of mice in the AAV-SNCA group compared to the AAV-NC group (Figure 5A,B). However, there was no statistically significant difference observed in Choline acetyltransferase (ChAT), a specific marker for ChI (Figure 5A,C). ELISA results demonstrated a reduction in ACh content in the dorsomedial striatum of mice in the AAV-SNCA group compared with that in the AAV-NC group (Figure 5D). Additionally, there was a significant reduction in the expression levels of Snap25 and Syntaxin in the AAV-SNCA group compared with those in the AAV-NC group (Figure 5E–G). These results suggest that α-syn aggregation in the dorsomedial striatum can inhibit ACh release from ChI.

### 3.5. Attenuation of M1R Activation Caused by α-Syn Aggregation Contribute to Reversal Learning Deficits

**Figure 6:**
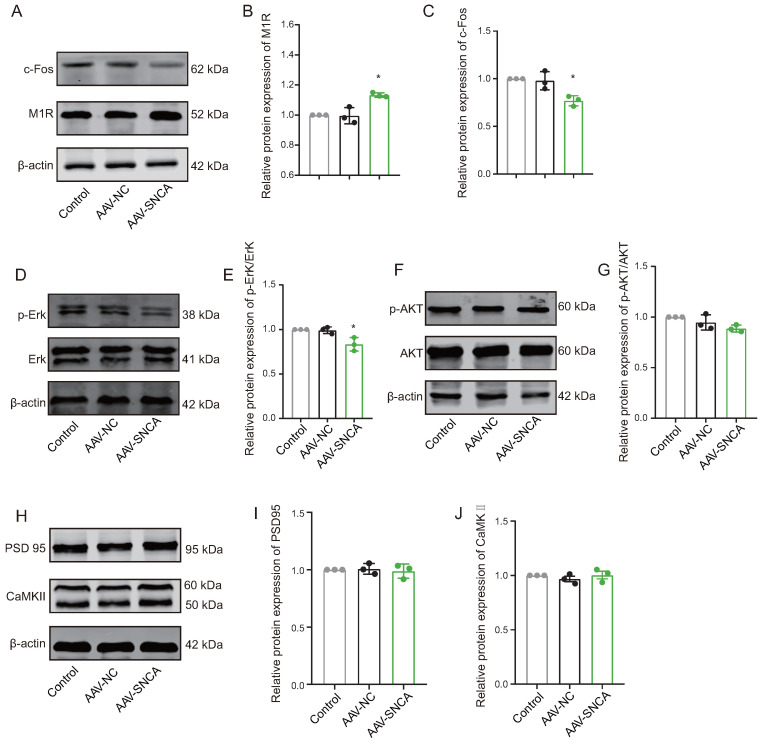
The aggregation of α-syn led to diminished activation on indirect pathway spiny projection neurons (iSPNs). (**A**–**C**) WB results and statistical analysis of c-Fos and M1R in the dorsomedial striatum. (**D**,**E**) The WB results were obtained for phosphorylated Erk (p-Erk), total Erk, and the ratio of p-Erk to Erk expression. (**F**,**G**) The WB results were obtained for phosphorylated AKT (p-AKT), total AKT, and the ratio of p-AKT to AKT expression. (**H**–**J**) WB results and statistical analysis of PSD 95 and CaMKII in the dorsomedial striatum. (*n* = 3, * *p* < 0.05 vs. AAV-NC group).

Reversal learning has been shown to be impaired by the inhibition of M1R expressed on the striatal indirect pathway spiny projection neurons (iSPN). In this study, compared to the AAV-NC group, Western blotting analysis revealed a significant increase in M1R expression levels (Figure 6A,B), while the expression levels of c-Fos (Figure 6A,C) and p-Erk/Erk (Figure 6D,E) were significantly decreased in the dorsomedial striatum of the AAV-SNCA group. Furthermore, there was no statistically significant difference in the expression levels of p-AKT/AKT, PSD 95, and CaMKII (Figure 6F–J). These results may suggest reduced activation of the iSPN by striatal α-syn aggregation, but are contradicted by an increase in M1R expression. M1R can be activated by acetylcholine (ACh). We speculate that α-syn aggregation results in decreased ACh, leading to diminished activation of M1R; consequently, there could be a compensatory increase in M1R expression.

**Figure 7:**
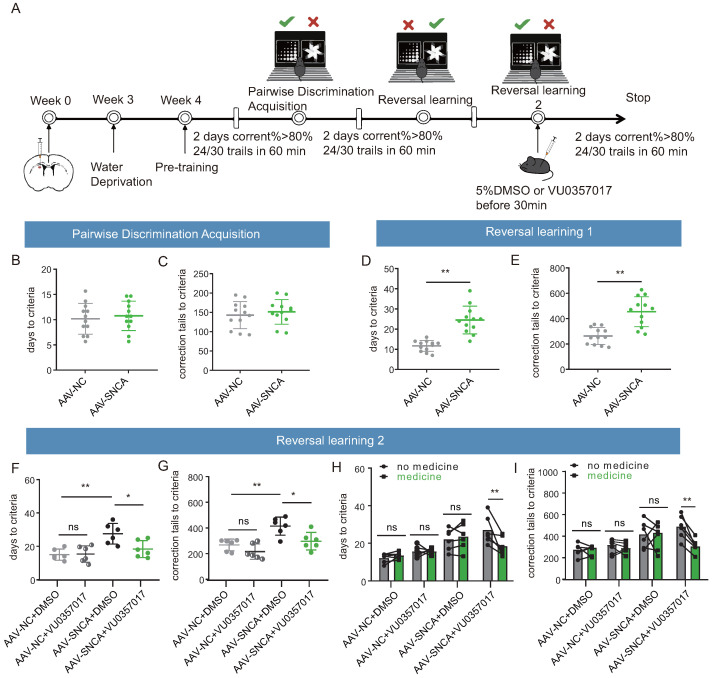
M1R activation mitigates the reversal learning deficits induced by α-syn aggregation in mice. (**A**) Experimental flow chart of drug administration and small animal touchscreen systems. (**B**,**C**) Statistical analysis of the time required and the number of errors corrected to achieve the criterion in the pairwise discrimination learning phase (*n* = 12 mice). (**D**,**E**) Statistical analysis of the time required and the number of errors corrected to achieve the criterion in the first reversal learning phase (*n* = 12 mice, ** *p* < 0.01 vs. AAV-NC group). (**F**,**G**) Statistical analysis of the time required and the number of errors corrected to achieve the criterion in the second reversal learning phase (*n* = 6 mice, * *p* < 0.05, ** *p* < 0.01 vs. AAV-SNCA + DMSO group); however, there was no significant difference between AAV-NC + DMSO and AAV-NC + VU0357017 groups. (**H**,**I**) Before and after intraperitoneal injection of M1R activator, statistical analysis of the time required and the number of errors corrected to achieve the criterion in the second reversal learning phase. A significant reduction in both the time required to achieve the criterion and the number of error corrections in the mice of the AAV-SNCA group were observed after intraperitoneal injection of U0357017 (** *p* < 0.01), whereas such effects were conspicuously absent in the AAV-NC group. Additionally, no significant differences were observed in these parameters in the two groups (AAV-NC and AAV-SNCA groups) before and after intraperitoneal injection of DMSO.

To confirm the role of M1R in reversal learning deficits caused by α-syn aggregation in the dorsomedial striatum, an activator of M1R (VU0357017) was co-administered in this study, followed by a visual discrimination reversal test to assess the inverse learning ability of each group of mice (Figure 7A). During the paired discrimination learning phase, the results of the small animal touch screen system were consistent with Figure 4(F2,F3), indicating that the initial learning ability of the mice remained unaffected by α-syn aggregation (Figure 7B,C). Similarly, during the reversal learning phase, the results in Figure 7D,E remained consistent with Figure 4G2,G3, showing that striatal α-syn aggregation leads to reversal learning deficits. Subsequently, a second reversal learning test was conducted following intraperitoneal injection of DMSO or VU0357017. The findings demonstrated a significant reduction in both the time required to achieve the criterion and the number of error corrections in the mice of the AAV-NC + DMSO and AAV-SNCA + VU0357017 groups, compared to those of the AAV-SNCA + DMSO group; however, there was no significant difference between AAV-NC + DMSO and AAV-NC + VU0357017 groups (Figure 7F,G). Further analysis of self-comparison using data from Figure 7D–G showed a significant reduction in both the time required to achieve the criterion and the number of error corrections in the mice of the AAV-SNCA group after intraperitoneal injection of VU0357017, whereas such effects were conspicuously absent in the AAV-NC group (Figure 7H,I). Additionally, no significant difference was observed in these parameters in the two groups before and after intraperitoneal injection of DMSO (Figure 7H,I). M1R activation alleviates reversal learning deficits caused by α-syn aggregation in the dorsomedial striatum.

## 4. Discussion

One prominent cognitive impairment seen in Parkinson’s Disease (PD) is a decline in cognitive flexibility, particularly in executive function, characterized by reversal learning deficits [2]. The primary pathological hallmark of PD is the depletion of dopamine in the nigrostriatal pathway. Levodopa, an exogenous dopamine drug, is commonly employed for symptomatic treatment [21]. However, clinical studies indicate that levodopa therapy does not ameliorate reversal learning ability in PD patients [4]. Consequently, this study focuses on the cholinergic pathway within the dorsomedial striatum, closely associated with reversal learning.

The MPTP subacute model stands as a classic PD research model, replicating PD pathology including dopaminergic neuron degeneration and α-syn aggregation [22]. While executive dysfunction is well-documented in MPTP-induced PD models [23], the presence of reversal learning deficits remains unclear. Herein, we establish a PD mouse model using MPTP subacute modeling. Our findings reveal that PD mice exhibit diminished reversal learning scores, often lingering in the quadrant previously housing the platform, as determined through a modified water maze experiment assessing reversal learning. These results provide preliminary evidence of impaired reversal learning ability in PD mice. Additionally, we observe a significant increase in phosphorylated α-syn (p-α-syn) levels in the dorsomedial striatum of PD mice, accompanied by α-syn aggregation. Given the positive correlation between α-syn aggregation and cognitive dysfunction, correlation analysis demonstrates a negative association between p-α-syn expression and reversal learning scores in PD mice, consistent with previous research and further suggesting a positive correlation between reversal learning deficits and α-syn aggregation.

The dorsomedial striatum emerges as a crucial brain region for reversal learning, with selective damage leading to impaired reversal learning ability in rats [5]. Optogenetic inhibition of indirect pathway spiny projection neurons (iSPNs) in the dorsomedial striatum induces reversal learning deficits in mice [6]. iSPNs in this region express dopamine D2 receptors and M1 muscarinic receptors (M1R) and receive regulatory input not only from nigral dopamine but also from cholinergic interneurons (ChIs) within the striatum [12,24]. Although both dopaminergic and cholinergic systems are implicated in executive dysfunction, dopamine loss alone does not decrease reversal learning ability [3,25], suggesting a pivotal role for cholinergic regulation of iSPNs within the striatum in reversal learning. Literature suggests that reversal learning is accompanied by increased ACh levels in the dorsomedial striatum [9], while clinical studies demonstrate cholinergic neuron loss in the basal ganglia of PD patients, closely associated with cognitive decline [26]. Additionally, pathological α-syn deposition in cholinergic fibers is observed in brain tissue slices from PD patients [17]. In our study, induction of α-syn aggregation in the dorsomedial striatum of mice results in a decrease in reversal learning ability despite equivalent initial learning levels, alongside reduced ACh content in the dorsomedial striatum. While alterations in ACh levels in the dorsomedial striatum may not impact initial learning ability [27], they play a critical role in behavioral changes during reversal learning, as supported by our findings [28].

ChI in the dorsomedial striatum serves as the primary source of ACh [12], expressing NR2D-containing NMDA receptors (NMDAR) and receiving glutamatergic projections from the thalamic parafascicular nucleus, thereby releasing ACh and activating M1 muscarinic receptors (M1R) on iSPN [12]. NR2D expression is exclusive to ChI within the striatum [29]. Literature also suggests that α-syn aggregation can downregulate the membrane expression of NMDAR [18]. In our study, induction of α-syn aggregation in the dorsomedial striatum led to decreased NR2D expression, while Choline Acetyltransferase (ChAT), a specific marker of ChI, remained relatively unchanged, indicating that α-syn aggregation downregulates NR2D expression without significantly affecting ChI survival. Additionally, expression of vesicle transport proteins Snap25 and Syntaxin, associated with neurotransmitter release, decreased, suggesting inhibited ACh release. These findings, combined with previous ELISA results of ACh levels, suggest that α-syn aggregation in the dorsomedial striatum inhibits ChI release by downregulating NR2D.

M1 muscarinic receptors (M1R) are the primary ACh receptors expressed on iSPNs in the dorsomedial striatum [8]. M1R, a G-protein coupled receptor, upon activation, promotes iSPN excitation by reducing dendritic K+ currents [30]. Studies have demonstrated that inhibition of M1R in the dorsomedial striatum leads to decreased Erk phosphorylation levels [31]. In our study, reversal learning deficit mice induced by α-syn aggregation in the dorsomedial striatum exhibited increased M1R expression, but decreased Erk phosphorylation levels and c-Fos expression, suggesting that M1R expression may increase in an attempt to maintain normal activity, yet fails to counteract the weakened iSPN activation caused by reduced ACh. Furthermore, expression of PSD95 and CaMKII, associated with iSPN synaptic plasticity, remained relatively unchanged, indicating no significant effect on iSPN synaptic plasticity, consistent with previous research [32]. Previous studies have demonstrated that injection of M1R inhibitors into the dorsomedial striatum impairs reversal learning in mice [15]. Building upon the reversal learning deficits induced by α-syn aggregation in the dorsomedial striatum, our study found that activation of M1R can mitigate these deficits when combined with M1R agonists, aligning with previous findings. Furthermore, our findings suggest that the effect of M1R agonists is not significant in the absence of reversal learning deficits. This may be due to the fact that reversal learning is more sensitive to acetylcholine, whereas the effect of M1R agonists on facilitating reversal learning is only evident when acetylcholine levels are reduced. Hence, it is hypothesized that α-syn aggregation in the dorsomedial striatum inhibits ACh release, weakens iSPN activation via M1R, and results in reversal learning deficits.

## 5. Conclusions

**Figure 8:**
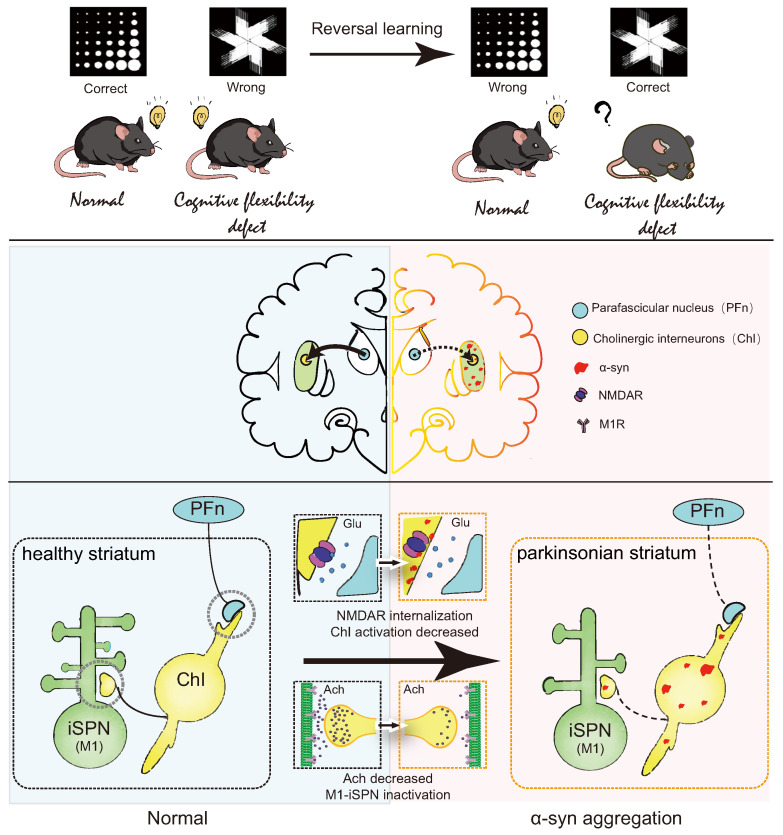
Summary diagram.

In summary, this study elucidates reversal learning deficits in PD model mice, closely linked to α-syn aggregation in the striatum. It provides preliminary insights into the mechanism by which α-syn aggregation in the dorsomedial striatum downregulates NR2D expression on ChI, inhibits ACh release, weakens iSPN activation, and consequently leads to reversal learning deficits (Figure 8). This project advances our understanding of the connection between α-syn aggregation and reversal learning, offering novel avenues for investigating PD with cognitive impairment and providing experimental evidence for the treatment of reversal learning deficits.

## Data Availability

The original contributions presented in the study are included in the article/Supplementary Material, further inquiries can be directed to the corresponding authors.

## Supplementary Materials

The following supporting information can be downloaded at: https://www.mdpi.com/article/10.3390/biomedicines12081634/s1, Table S1. The ELISA results demonstrate the quantification of dopamine levels in the substantia nigra; Figure S1. The ELISA results demonstrate the quantification of dopamine levels in the substantia nigra (*n* = 8, * *p* = 0.019 vs. Control).
