# Supplementary material for: The Aggregation of α-Synuclein in the Dorsomedial Striatum Significantly Impairs Cognitive Flexibility in Parkinson’s Disease Mice
Source: Biomedicines. 2024 Jul 23;12(8):1634. doi: 10.3390/biomedicines12081634 (PMC11351470; doi:10.3390/biomedicines12081634)
Supplement: Supplementary file 1 [file biomedicines-12-01634-s001.zip › biomedicines-3065472-supplementary.pdf]

Supplementary Material S1

Table S1. The ELISA results demonstrate the quantification of dopamine levels in the substantia nigra

group	DA (pg/mL)
Control	153.9575 ± 7.08762
MPTP	144.8875 ± 6.61258*

*P = 0.019 *vs* Control (n = 8)

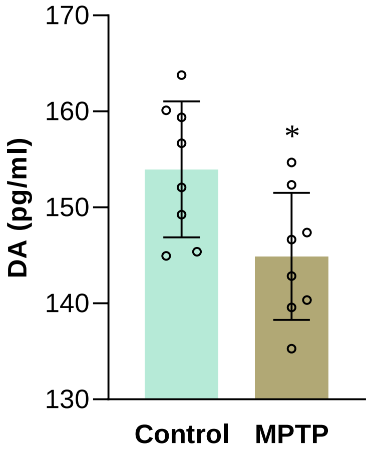

Figure S1. The ELISA results demonstrate the quantification of dopamine levels in the substantia nigra (n = 8, *P = 0.019 *vs* Control)

Supplementary Material S2

REAGENT ＆ VIRUS		SOURCE	IDENTIFIE	Notes
anti-AKT	56kDa	Proteintech	Cat# 10176-2-AP, RRID:AB_2224574	WB 1:5000 IF 1:500
anti-a-syn	14kDa	Abcam	Cat# ab138501, RRID:AB_2537217	WB 1:5000 IF 1:500
anti-CaMKII	50kDa	Abcam	Cat# ab181052; RRID:AB_2891241	WB 1:1500
anti-c-fos	62kDa	Santa Cruz	Cat# sc-166940 AF647, RRID:AB_2943286	WB 1:500 IF 1:200
Anti-Choline Acetyltransferase	82kDa	Abcam	Cat# ab181023, RRID:AB_2687983	WB 1:5000 IF 1:100
anti-ERK1/2	42-44kDa	Sigma-Aldrich	Cat# M5670, RRID:AB_477216	WB 1:20000
Anti-GluN2D	144kDa	Thermo Fisher Scientific	Cat# PA5-101608, RRID:AB_2851042	WB 1:1000 IF 1:500
anti-M1R	52kDa	Santa Cruz	Cat# sc-365966; RRID:AB_10847359	WB 1:500 IF 1:200
anti-p-AKT	60kDa	Affinity	Cat# AF0016, RRID:AB_2810275	WB 1:1000 IF 1:500
anti-Phospho-alpha Synuclein (Ser129)	18kDa	Thermo Fisher Scientific	Cat# PA5-37740, RRID:AB_2554348	WB 1:1000 IF 1:750
anti-p-ERK1/2	38-43kDa	Proteintech	Cat# 28733-1-AP; RRID:AB_2881202	WB 1:5000
Anti-PSD-95	95kDa	CST	Cat# 3450S; RRID: AB_2292883	WB 1:1000 IF 1:200
anti-SNAP25	25kDa	Santa Cruz	Cat# sc-20038; RRID:AB_628264	WB 1:500 IF 1:200
Anti-Syntaxin	35kDa	Santa Cruz	Cat# sc-20036, RRID:AB_628316	WB 1:5000
Anti-TH	60kDa	Santa Cruz	Cat# sc-374048, RRID:AB_10917743	WB 1:1000 IF 1:200
Anti-β-actin	42kDa	Proteintech	Cat# 66009-1-Ig; RRID: AB_2782959	WB 1:20000
PFD-rAAV-SYN(BrainVTA) -SNCA(A53T)		BrainVTA	Cat# GT-0072	viral titre (5.63E + 12 vg/mL)
VU0357017（M1R agonist）		TargetMol	Cat# T3619	33 mg/mL (DMSO) ; 1.67 mg/mL in double-distilled water (5% DMSO) 20 mg/kg
